# Proof of concept that melanoma nuclear count compares favourably with the benchmark histological prognostic feature, Breslow thickness

**DOI:** 10.1111/his.15300

**Published:** 2024-08-13

**Authors:** Charlotte Gurr, Mark Bamford, Nicola Oswald, Louisa Udensi, Christopher Ready, Kritika Gupta, Tiffany Buhagiar, Gerald Saldanha

**Affiliations:** ^1^ University of Leicester Leicester UK; ^2^ University Hospitals of Leicester NHS Trust Leicester UK

**Keywords:** biomarker, cancer, dermatology, melanoma, prognosis

## Abstract

**Aims:**

Breslow thickness (BT) is the most important histological prognostic feature for melanoma prognosis, but it only captures tumour size in one dimension. Adding a further measurement in a different axis has been shown to improve prognostic value. It seems reasonable that further prognostic value could be obtained by estimating the number of invasive melanoma cells using nuclear count. The aim of this study was to show proof of concept that nuclear count has prognostic value independent of BT.

**Methods and results:**

Melanoma cell nuclei were labelled with SRY‐related HMG‐box 10 (SOX10) protein, the sections scanned and StarDist machine‐learning algorithm used to count nuclei in 102 cases of primary cutaneous melanoma. Prognostic value was assessed using survival analyses. Nuclear count correlated strongly with T category, BT and calculated tumour area (each *P* < 0.001), suggesting that it was a valid marker of melanoma burden. Nuclear count was a predictor for overall survival in univariable analysis [hazard ratio (HR) = 2.25, confidence interval (CI) = 1.66–3.06, *P* < 0.001] and multivariable analysis (HR = 2.60, CI = 1.59–4.24, *P* < 0.001). BT and ulceration were significant in univariable analyses, but not in multivariable models with nuclear count. Models containing nuclear count showed the best fit. Similar results were seen for melanoma‐specific and metastasis‐free survival. Nuclear count was able to stratify melanomas within a given T stage.

**Conclusions:**

This study demonstrated proof of concept that counting melanoma nuclei may be an improved measure of invasive tumour burden compared to BT. Future studies will need to refine methods of nuclear detection and also to confirm its prognostic value.

## Graphical abstract

Breslow thickness (BT) is the most important histological prognostic feature for melanoma prognosis. A count of invasive melanoma cell nuclei may refine and improve BT. This study demonstrates proof of concept that this proposal is correct.

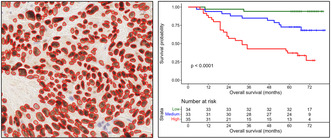

**Abbreviations**
AIC: Akaike information criterion
ANOVA: Analysis of variance
BT: Breslow Thickness
CPH: Cox proportional hazards regression
CTA: Calculated tumour area
DAB: 3,3′‐diaminobenzidine
IQR: Interquartile range
MFS: Metastasis‐free survival
MSS: Melanoma specific survival
NC: Nuclear count
OS: Overall survival
SOX10: SRY‐related HMG‐box 10
UICC: Union for International Cancer Control
WSI: Whole slide image

## Introduction

Cutaneous melanoma is an aggressive disease, but most patients are cured when treated early in their clinical course.^1^ Predicting the risk of disease progression and death is crucial for stratifying patients, the key histological feature for this being Breslow thickness (BT),^2^ which is the vertical depth in millimetres that the tumour has invaded below the epidermal stratum corneum. It is the cornerstone for specifying the T category in the TNM staging system,^3^, ^4^, ^5^ which is used to choose re‐excision margins and select for sentinel node biopsy.

BT is surrogate for the total burden of invasive melanoma cells. An exhaustive count of invasive melanoma cells would be challenging, requiring the whole tumour to be serially sliced, every slice embedded and invasive tumour cells counted in each with morphometric three‐dimensional reconstruction, taking into account slice thickness. In addition, it would be necessary to ensure that *in‐situ* melanoma cells were carefully eliminated from the count. In this light, BT is an effective compromise. Nevertheless, BT is only a one‐dimensional distance. This means that a prognostic distinction between two melanomas with the same BT could be made by incorporating a second dimension in a different axis; i.e. BT could be refined.

Some authors of the present study have investigated BT refinement. Thus, the local density of invasive cells at the position of BT measurement was determined using ‘Breslow density’, which was combined with BT into a ‘targeted burden score’.^6^, ^7^ The width of invasive tumour measured parallel to the epidermis was investigated for its prognostic value.^8^ Furthermore, a simple semi‐quantitative method to estimate the total area occupied by invasive cells on the same histological slide that BT was measured was investigated using ‘calculated tumour area’ (CTA).^9^ All these features showed prognostic distinction between two tumours with the same BT, were simple to measure and therefore could easily be adopted in clinical practice if confirmed by future studies.

A shortfall of all these metrics is that they do not provide an actual cell count. This would be potentially better, because it becomes closer to reflecting the value we care about: the total number of invasive melanoma cells. A manual count of cells in the transverse section where BT was measured would be laborious for histopathologists in daily practice. However, an automated count on scanned whole slide images (WSIs) is possible. WSIs are not universally used, but some National Health Service (NHS) laboratories have adopted them,^10^, ^11^ their popularity is likely to increase and they show equivalence to glass slides for diagnosis.^12^ Image analysis of WSIs, potentially with machine‐learning algorithms, may yield novel biomarkers in future.^13^

This study's aim was to show proof of concept that a measure of the total number of invasive melanoma cells would be a better surrogate of invasive burden than BT and hence have independent prognostic value, thus refining BT. A surrogate for melanoma count was chosen; namely, SRY‐related HMG‐box 10 (SOX10) protein‐positive melanoma nuclei, determined by image analysis of WSIs. SOX10 is a sensitive and relatively specific marker for melanoma nuclei. The aim was not to develop a definitive method for semi‐automated nuclear counting, but rather to develop a method minimally robust enough to show proof of concept that a definitive method could be developed in the future, and to show sufficient prognostic validity in comparison to BT, such that there would be evidence to pursue this in future.

## Materials and methods

### Patients and samples

Archival tissue use was approved by the East Midlands Research Ethics Committee (17/EM/0125). Retrospective melanoma cases with BT greater than 1.0 mm from the University Hospitals of Leicester (UHL) histopathology diagnostic archive were assessed sequentially from 1 December 2012 until 31 March 2014. A total of 150 cases were assessed, and after exclusions 102 were analysed (Supporting information, Figure S1). Baseline features are shown in Table 1 for all cases, also broken down by nuclear count. Patients classified as UICC version 8 stage III were all due to microscopic satellite deposits without regional node disease (pN1c) at diagnosis.

**Table 1:** Patient demographics

	Overall	Nuclear count low	Nuclear count med	Nuclear count high	*P*
*n*	102	34	33	35	
Nuclear count (1000s) (median [IQR])	30.92 [18.25, 104.71]	12.95 [7.24, 18.03]	30.85 [27.73, 48.01]	139.68 [104.45, 196.63]	NA
Age (median [IQR])	69.00 [55.00, 77.00]	63.50 [48.75, 73.50]	68.00 [55.00, 76.00]	74.00 [65.00, 84.50]	0.017
Sex, male (%)	58 (56.9)	16 (47.1)	17 (51.5)	25 (71.4)	0.093
Site (%)					
Acral	2 (2.0)	2 (5.9)	0 (0.0)	0 (0.0)	0.152
Head and neck	24 (23.5)	5 (14.7)	10 (30.3)	9 (25.7)	
Lower limb	28 (27.5)	13 (38.2)	5 (15.2)	10 (28.6)	
Trunk	26 (25.5)	7 (20.6)	8 (24.2)	11 (31.4)	
Upper limb	22 (21.6)	7 (20.6)	10 (30.3)	5 (14.3)	
Breslow thickness (median [IQR])	2.25 [1.30, 3.88]	1.30 [1.12, 1.50]	1.90 [1.50, 2.50]	4.10 [3.40, 5.50]	< 0.001
Ulcer, yes (%)	38 (37.3)	4 (11.8)	11 (33.3)	23 (65.7)	< 0.001
Mitoses (median [IQR])	3.00 [1.00, 4.75]	1.00 [1.00, 2.00]	3.00 [2.00, 6.00]	4.00 [3.00, 7.50]	< 0.001
Microsatellites, yes (%)	9 (8.8)	1 (2.9)	2 (6.1)	6 (17.1)	0.091
UICC version 8 (%)					
IB	36 (35.3)	24 (70.6)	12 (36.4)	0 (0.0)	< 0.001
IIA	29 (28.4)	8 (23.5)	15 (45.5)	6 (17.1)	
IIB	18 (17.6)	1 (2.9)	4 (12.1)	13 (37.1)	
IIC	10 (9.8)	0 (0.0)	0 (0.0)	10 (28.6)	
III	9 (8.8)	1 (2.9)	2 (6.1)	6 (17.1)	

IQR, interquartile range; UICC, Union for International Cancer Control.

### Immunohistochemistry

Haematoxylin and eosin (H&E)‐ and SOX10‐stained sections were prepared as per standard protocol in the UHL diagnostic histopathology laboratory. Briefly, mouse monoclonal antibody to SOX10 (Abcam, Cambridge, UK) was used, stained on the Dako OMNIS automated stain machine (Agilent, Cheadle, Chesire, UK) using the Agilent Envision FLEX+ system at 1:100 dilution with horseradish peroxidase and 3,3′‐diaminobenzidine (DAB) as a brown chromogen. Sections were counterstained with haematoxylin.

### Slide scanning and image pre‐processing

The SOX10‐stained sections were scanned at ×200 using the Hamamatsu NanoZoomer S210 (Welwyn Garden City, UK) with resolution of 0.4415 μm per pixel and saved as normalised differential phenology index (NDPI) files. These were analysed using QuPath version 0.4.2.^14^ For each, the stain vector was manually adjusted, as described in the QuPath manual. Next, it was necessary to select regions of WSI for the StarDist algorithm. First, the chosen section was surrounding by a hand‐drawn annotation. To narrow this region to areas containing melanoma cells, QuPath was used to more finely identify areas containing SOX10‐positive nuclei using a pixel classifier based on a DAB stain intensity threshold. This classifier was built using one WSI and then applied to all other WSIs. Areas of *in‐situ* melanoma and stromal melanin were manually removed (Supporting information, Figure S2), as were areas of benign naevus.

### Nuclear counting using StarDist

StarDist was originally developed as a python package,^15^, ^16^ but we used a Java Groovy script adapted for use in QuPath. We would have preferred to use red detection, but the code was developed to detect brown DAB‐stained nuclei, originally developed by Zaidi and adapted for this study.^17^ An important element is the ‘param channel’, which refers to the deconvoluted colour channel StarDist uses for nuclear detection. Channel 2 is the deconvoluted DAB channel from whose pixels StarDist infers nuclei. The nuclear counts were split into tertiles for low, medium and high categories.

### Statistical analysis

Statistical analysis was conducted using R version 4.3.1^18^ in RStudio,^19^ with *P* < 0.05 regarded as significant in two‐tailed tests. Baseline statistics for numerical variables consisted of median and interquartile range (IQR) and for categorical variables, counts and percentages. To determine the association between nuclear count tertiles and continuous baseline melanoma variables a Kruskal–Wallace test was performed, and a χ^2^ test was used for categorical variables. To compare nuclear count as a continuous variable with BT and CTA, a Spearman's rank correlation test was used and with T category a Kruskal–Wallace test. The survival analyses included three endpoints modelled using Cox proportional hazards (CPH) regression: overall survival (OS), melanoma‐specific survival (MSS) and metastasis‐free survival (MFS). OS was the primary outcome. An event was death from any cause and patients remaining alive were censored. For MSS, an event was death due to melanoma with censoring when the patient died from another cause or remained alive. For MFS, an event was first clinical metastasis, either regional or distant, with censoring if the patient died without metastasis or remained alive. Microscopic satellites found at primary or wide local excision were ignored in this analysis. Sentinel node biopsy was not performed at UHL in the recruitment period, and these data were not available for any patient. Melanoma follow‐up and imaging was according to standard United Kingdom guidelines at the time. Median follow‐up was 65 months. Survival version 2.43–3^20^ and survminer version 0.4.5^21^ R packages were used for CPH regression models. The proportionality assumption was checked with plots of scaled Schoenfeld residuals against transformed time and a goodness‐of‐fit test. Proportionality was not violated. CPH model fit was compared using the Akaike information criterion (AIC),^22^ where ΔAIC of > 4 between each model indicates support for the model with the lower value and greater than 10 indicates very strong support. BT and log‐nuclear count (which was entered into regression models) were correlated, but the variance inflation factor (VIF) was 3.3, suggesting no serious issue with collinearity. Multivariable survival analysis was limited to three predictor variables to ensure adequate power because the primary outcome, OS, had 37 events and 10 events per outcome is regarded as minimally adequate.^23^, ^24^, ^25^ Remark guideline adherence is shown in Supporting information, Table S1.

## Results

### Patient demographic features

Patient demographics are shown in Table 1, being as expected from a UK hospital for patients with melanomas > 1 mm.^26^ All the features except site, sex and microscopic satellites were associated with nuclear count. Sex was nearly significant and there were only nine microscopic satellites in total, so the power to identify an effect was low.

### Method validation

**Figure 1:**
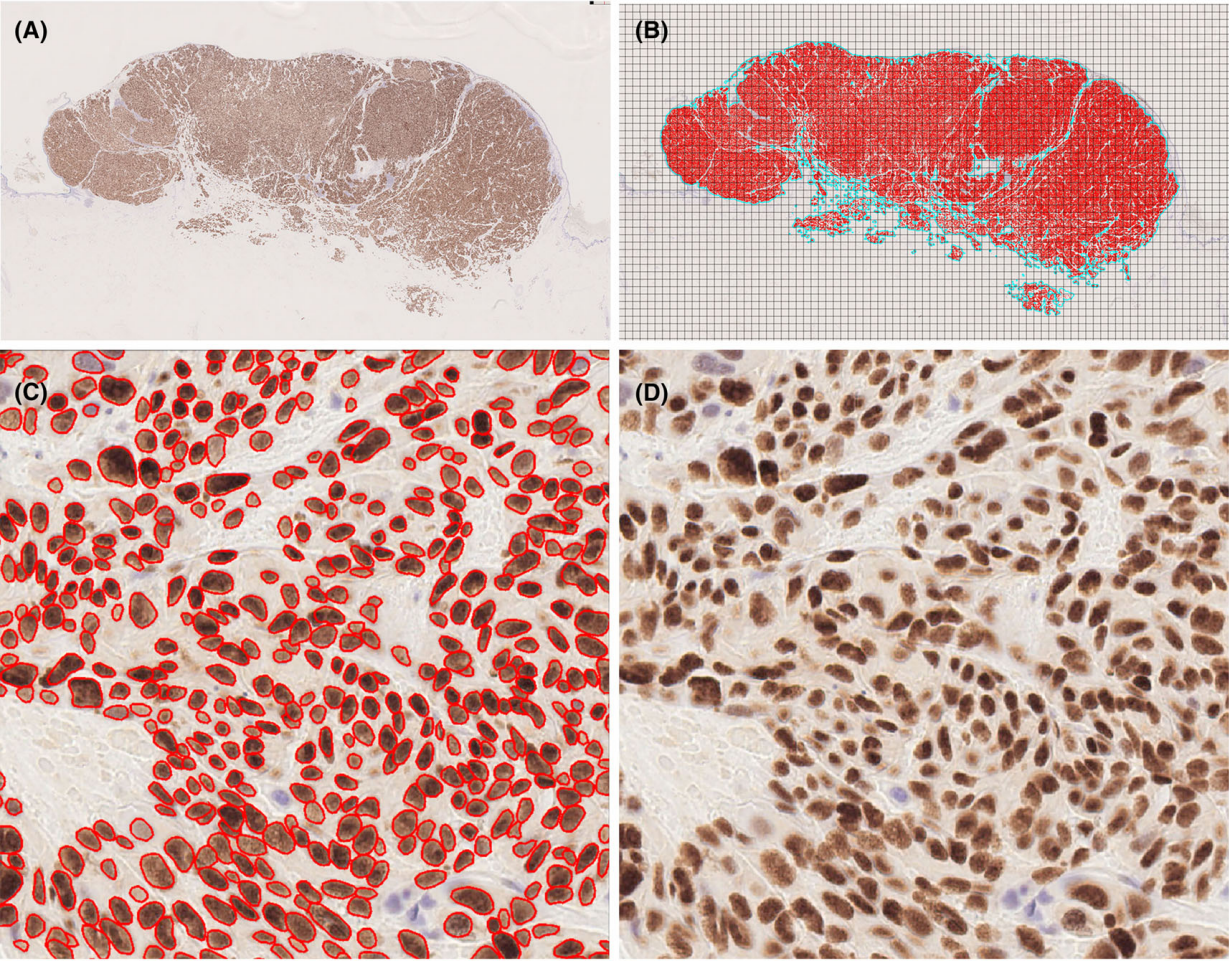
An example WSI without (**A**) and with annotations plus grid overlay (**B**). Close‐up of a single 0.25 × 0.25‐mm grid box with (**C**) and without (**D**) and annotations. [Color figure can be viewed at wileyonlinelibrary.com]

**Figure 2:**
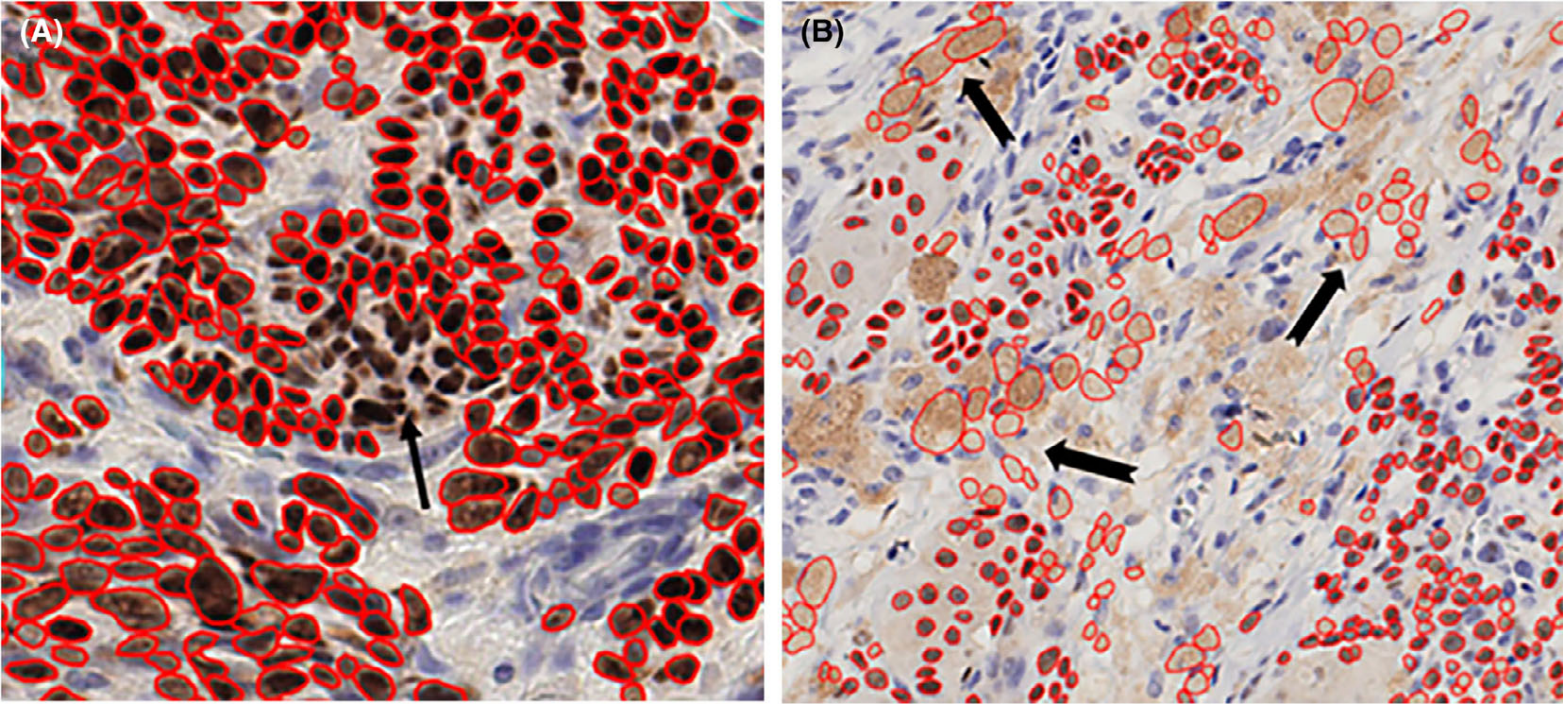
False‐negative annotation (**A**) in areas of crowded and overlapping nuclei and of false‐positive annotations due to melanin. [Color figure can be viewed at wileyonlinelibrary.com]

**Figure 3:**
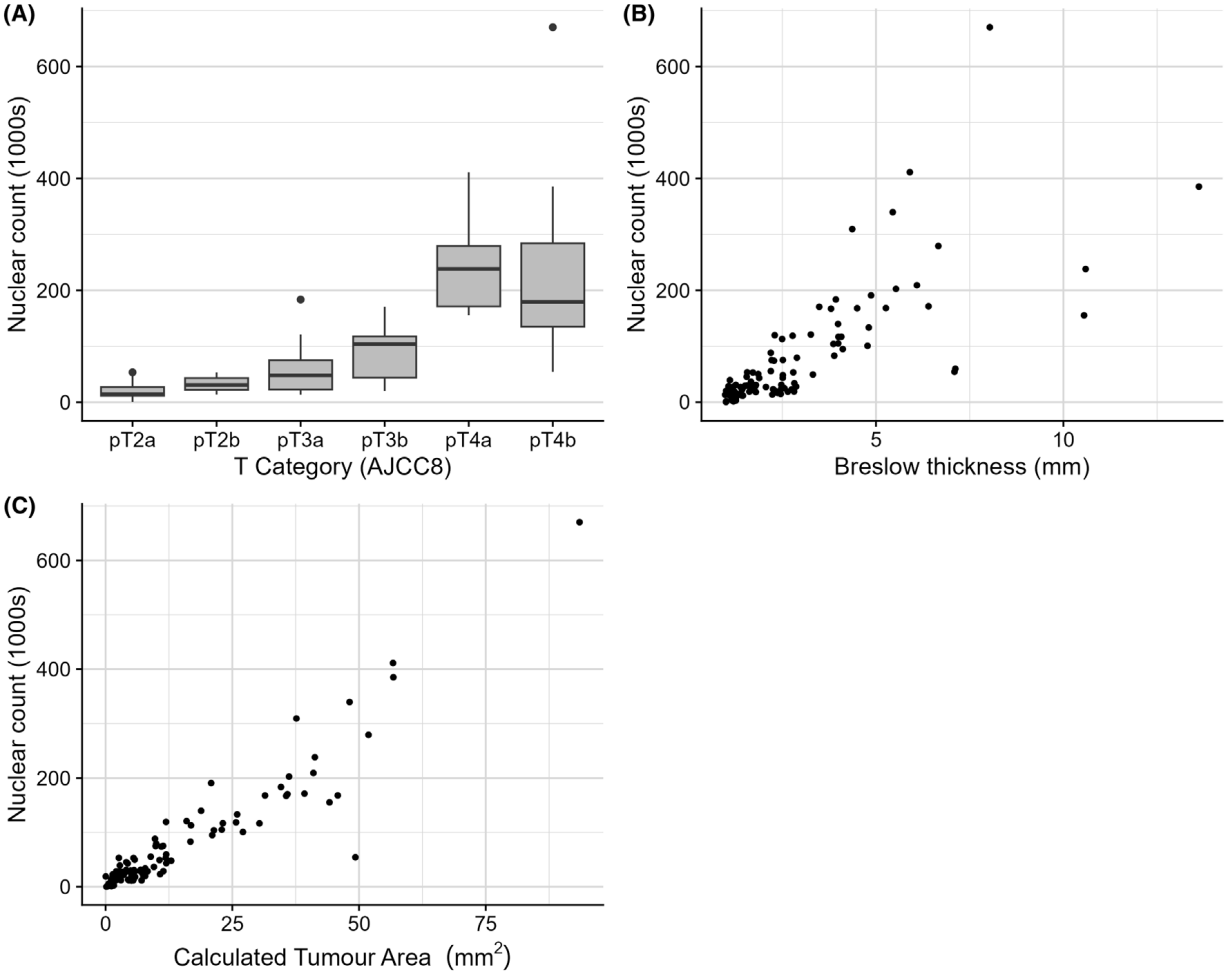
Nuclear count plotted against T category (**A**), Breslow thickness (BT) (**B**) and calculated tumour area (CTA) (**C**).

To verify that the algorithm correctly detected melanoma nuclei, up to 10 random areas from each WSI (0.25 × 0.25 mm) were inspected for accuracy of StarDist nuclear annotations, as seen in Figure 1. Important causes of inaccurate annotation were false‐negatives due to crowded/overlapping nuclei and false‐positives caused by melanin pigment in stromal cells. Examples are shown in Figure 2. For all cases, heavily pigmented stromal areas were manually excluded from the selected areas for StarDist analysis, minimising the problem. The areas of false‐negatives were generally a tiny fraction of all the nuclei identified per case, given that the median count was 30 000 nuclei. The validity of raw nuclear counts as an invasive burden marker was tested by assessing its correlation with T category, BT and CTA. Figure 3 shows that each had a significant relationship (*P* < 0.001).

### Nuclear count prognostic value

**Figure 4:**
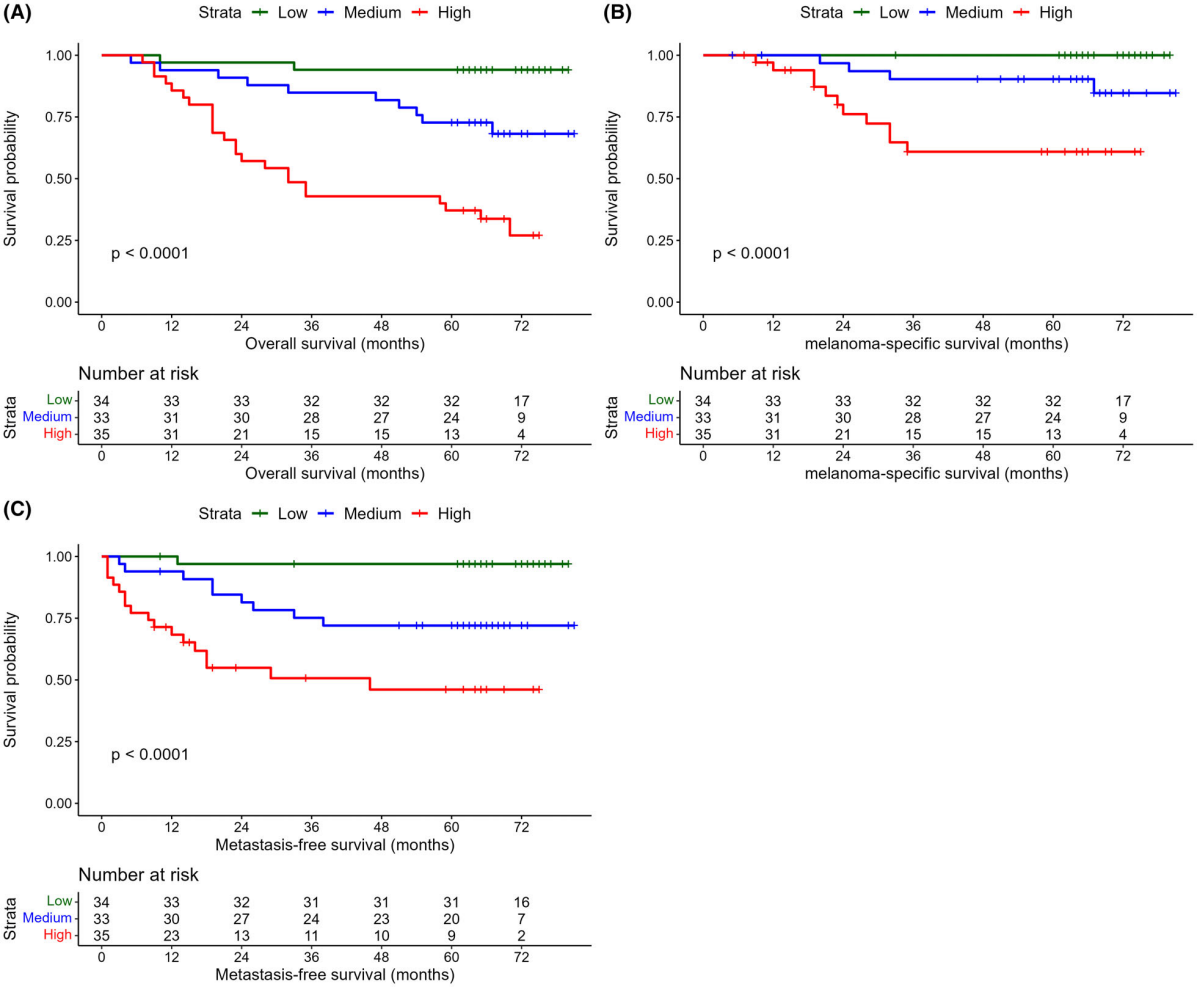
Kaplan–Meier plots. Nuclear count was split into tertiles and curves demonstrate overall survival (**A**), melanoma‐specific survival (**B**) and metastasis‐free survival (**C**). Each show highly significant separation. [Color figure can be viewed at wileyonlinelibrary.com]

The univariate effect of nuclear count on melanoma outcome was assessed using Kaplan–Meier plots and log‐rank tests (Figure 4). The 5‐year OS for low, medium and high nuclear count were 94, 73 and 37%, respectively. The corresponding figures for melanoma‐specific survival were 100, 90 and 61% and for metastasis‐free survival were 97, 72 and 46%. To assess the adjusted effect of nuclear count on melanoma survival, CPHs regression was used. Nuclear count was severely right‐skewed, so it was log‐transformed prior to entry into the model. The results are summarised in Table 2.

**Table 2:** Cox proportional hazards regression models

	HR (univariable)	HR (multivariable)
Overall survival		
Breslow thickness	1.21 (1.10–1.33, *P* < 0.001)	0.93 (0.78–1.12, *P* = 0.435)
Ulcer		
No	–	–
Yes	2.42 (1.25–4.68, *P* = 0.008)	0.97 (0.47–2.03, *P* = 0.941)
Nuclear count (log‐transformed)	2.25 (1.66–3.06, *P* < 0.001)	2.60 (1.59–4.24, *P* < 0.001)
Melanoma‐specific survival		
Breslow thickness	1.25 (1.10–1.42, *P* = 0.001)	0.89 (0.68–1.16, *P* = 0.380)
Ulcer		
No	–	–
Yes	3.91 (1.33–11.44, *P* = 0.013)	1.15 (0.35–3.75, *P* = 0.814)
Nuclear count (log‐transformed)	3.19 (1.90–5.36, *P* < 0.001)	4.00 (1.78–8.97, *P* = 0.001)
Metastasis‐free survival		
Breslow thickness	1.21 (1.09–1.34, *P* < 0.001)	0.97 (0.80–1.16, *P* = 0.713)
Ulcer		
No	–	–
Yes	4.19 (1.88–9.34, *P* < 0.001)	1.92 (0.79–4.65, *P* = 0.149)
Nuclear count (log‐transformed)	2.29 (1.61–3.24, *P* < 0.001)	2.17 (1.26–3.75, *P* = 0.005)

HR, hazard ratio.

The univariable effect of nuclear count on OS, MSS and MFS was significant, as were BT and ulcer. However, in a multivariable model containing all three features, only nuclear count was significant (*P* < 0.001), as shown in Table 2. The effect of nuclear count on model fit was assessed. The ΔAIC for OS and MSS revealed that the model with nuclear count was > 10 points lower, consistent with very strong support for the superiority of this model, while for MFS it was > 4 lower, indicating support. These data indicate that the model with nuclear count had the optimal fit (Table 3). This is corroborated by an analysis of variance (ANOVA) test for each outcome, showing that the addition of nuclear count resulted in a highly statistically significant improvement (Table 3).

**Table 3:** ΔAIC values for Cox proportional hazards models and ANOVA tests to assess statistical significance

Model variables	OS		MSS		MFS	
	ΔAIC	ANOVA	ΔAIC	ANOVA	ΔAIC	ANOVA
BT, Ulcer	14.8	—	11.6	—	6.6	—
BT, Ulcer, NC	0.0	*P* < 0.001	0.0	*P* < 0.001	0.0	*P* < 0.01

AIC, Akaike information criterion; MFS, metastasis‐free survival; MSS, melanoma‐specific survival; NC, nuclear count; OS, overall survival; ANOVA, analysis of variance.

**Figure 5:**
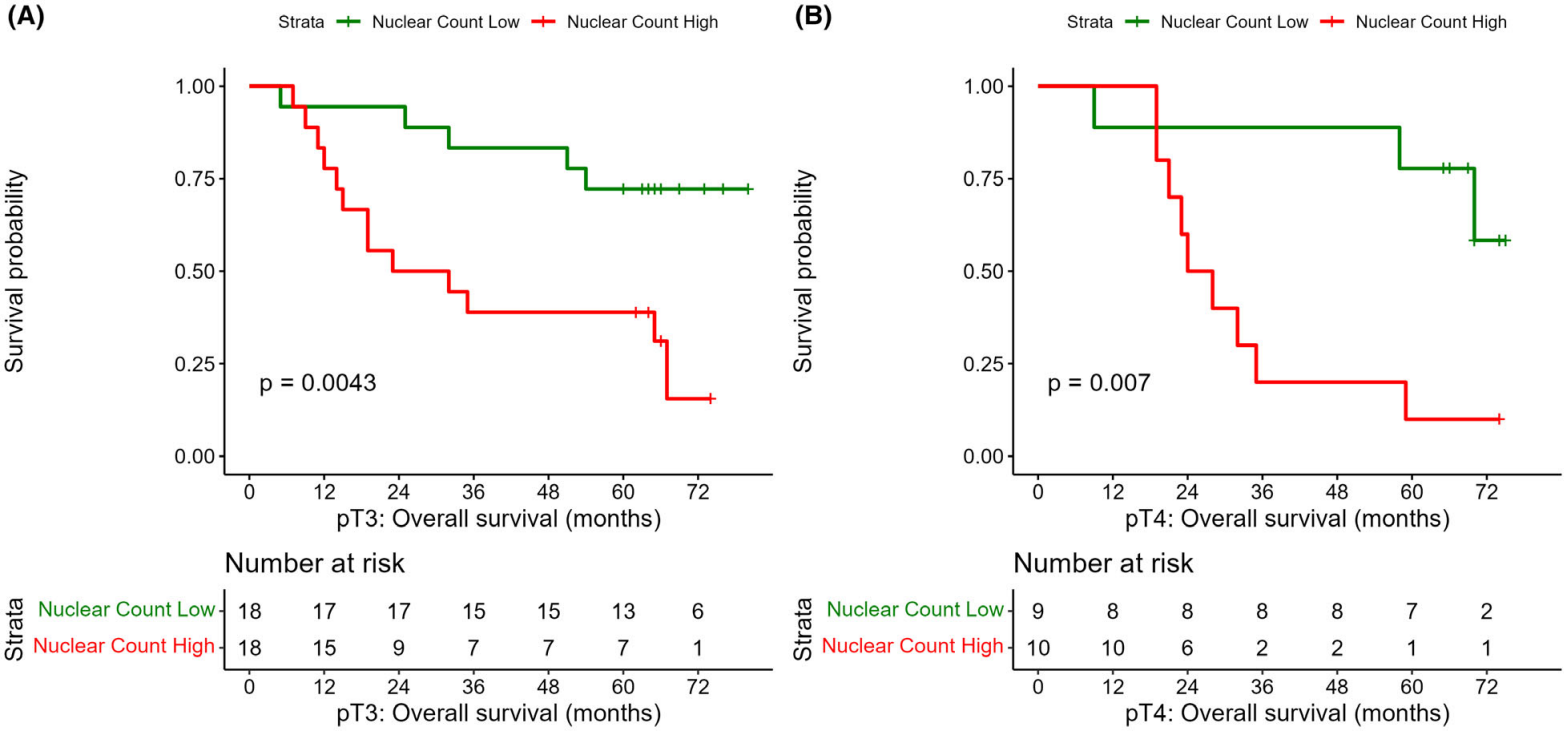
Kaplan–Meier plots of nuclear count split at median within pT3 (**A**) and pT4 (**B**) melanoma subsets, showing that nuclear count has potential to refine T stage category. [Color figure can be viewed at wileyonlinelibrary.com]

Finally, an assessment was made to indicate whether nuclear count could subclassify T category to add nuanced survival information. This was explored by creating subgroups of pT3 and pT4 melanomas, the only melanomas that had sufficient OS outcomes for analysis. Nuclear count was split at respective subgroup medians and Kaplan–Meier plots made, shown in Figure 5, demonstrating significant differences for pT3 and pT4. This suggests that nuclear count has potential as an adjunct to refine clinical T category.

## Discussion

This proof‐of‐concept study evaluated whether a count of invasive melanoma cell nuclei would be a clearer measure of invasive burden than BT. It was found that nuclear count correlated strongly with other measures of tumour burden, suggesting its validity. Furthermore, nuclear count was a predictor for survival in univariable and multivariable analysis, being clearer than both BT and ulceration. Nuclear count stratified melanomas within a given T category, showing its potential to refine stage. The population used was representative of melanomas greater than 1 mm in UK patients,^26^ and therefore the results may be applicable to patients of similar stages in future studies.

Measuring the prognostic value of total invasive melanoma cell burden has been attempted in terms of estimates of tumour volume,^27^, ^28^, ^29^ but is impractical for routine clinical practice. BT is a surrogate for this and is easily measured. However, it is limited by being measured in one dimension. Breslow also assessed tumour cross‐sectional area, but it was based on depth and macroscopic diameter,^2^ which would include *in‐situ* disease. Previously, some of the current authors looked at methods to refine BT using two‐dimensional features—namely, Breslow density^6^, ^7^ and CTA,^9^ finding strong prognostic value. In principle, nuclear count is a further improvement, because it estimates actual invasive cell numbers rather than a distance (BT), a local density (BD) or an area (CTA), and therefore is probably a truer surrogate of invasive cell numbers. Kaplan–Meier curves showed that a higher nuclear count was associated with a poorer prognosis for OS, MSS and MFS, also being significant in multivariable CPHs analysis. AIC and ANOVA tests to compare models showed that nuclear count improved model fit when added to BT and ulcer. Conversely, neither BT or ulcer were significant in multivariable analysis when nuclear count was a variable in the model.

This study required the use of WSI, which are not routinely used in most centres. Nevertheless, the future of pathology is likely to become increasingly digital. Some UK histopathology departments are preparing to digitise glass slides and have received government funding for investigation into the use of artificial intelligence.^30^ WSI and image analysis may allow a more accurate assessment of established histopathological features and offer potential to discover new prognostic features. This study demonstrates that development of a validated image analysis method to perform melanoma nuclear counting would be worthwhile. Even so, it must be acknowledged that nuclear count analysis would require additional time and resources for routine use.

As a proof‐of‐concept study, there are limitations. This is a small study, and method validation is needed. The exclusions were high. In particular, 17 cases were excluded due to high amounts of melanin, which causes false detections by the StarDist algorithm. This is because the QuPath script required a brown DAB chromogen. In future, the algorithm needs to be changed so it can be used with a red chromogen. Cases in which the transverse section where BT was measured was divided and placed into separate blocks were excluded. In future, we would include these. Having run StarDist multiple times on test regions it always produced the same result, but test reproducibility was not formally assessed because we regarded this study as proof of concept. Measures of test validity and reproducibility would be needed for a future definitive method, especially regarding the subjective manual parts of the process, such as choosing regions for stain vector correction, manual exclusion of stromal melanin and manual removal of *in‐situ* melanoma. The study patients were diagnosed from the beginning of 2012 to March 2014, with a median survival of 65 months, so changes in therapy could be a confounding feature. However, this would also be true for BT, against which we compared nuclear count. The lack of SLN biopsy in the study cohort indicates that time to metastasis would not be generalisable to institutions where SLN biopsy is used (at least with regard to the draining node basin). The method would not be suitable for cases with substantial co‐existent naevus. An algorithm that could use preferentially expressed antigen in melanoma (PRAME) or a person trained on the H&E features that could distinguish melanoma from naevus might help.

This study was powered to allow analysis of three independent variables in multivariate Cox regression, namely BT and nuclear count, together with ulceration: the one other histological variable used in TNM8 staging. This was deemed appropriate, as we wished to focus upon comparison of nuclear count and BT. In future studies, nuclear count will need to tested against a more complete list of confounding variables requiring a much larger sample, including baseline features such as age and mitoses, that were significantly associated with nuclear count in this study. If nuclear count is to be used as an adjunct to stratify patients for sentinel node biopsy it would be necessary to also include pT1 melanomas.

In summary, this study demonstrates proof of concept that counting melanoma nuclei on the same slide on which BT was measured is a novel prognostic feature, and is a more effective surrogate for invasive melanoma burden. In future, studies will need to explore ways to improve the method of nuclear detection and also to verify its prognostic value. These findings are of great relevance to melanoma clinical care, given the need to refine melanoma patient stratification. In this regard, nuclear count has potential to be developed as an adjunct to staging, especially if histology slide digitisation becomes more widely adopted.

## Conflicts of interest

The authors declare that they have no conflicts of interest.

## Supporting information


**Figure S1.** Selection of melanoma cases. Review point 1 entailed assessment of the original melanoma report and archival slides.
**Figure S2.** Top panel shows WSI with hand drawn selection in red.
**Table S1.** REMARK guideline features.

## Data Availability

The data underlying this article will be shared on reasonable request to the corresponding author.
