# Supplementary material for: Proof of concept that melanoma nuclear count compares favourably with the benchmark histological prognostic feature, Breslow thickness
Source: Histopathology. 2024 Aug 13;86(2):226–35. doi: 10.1111/his.15300 (PMC11649523; doi:10.1111/his.15300)
Supplement: Supplementary file 1 — Figure S1. Selection of melanoma cases. Review point 1 entailed assessment of the original melanoma report and archival slides. Figure S2. Top panel shows WSI with hand drawn selection in red. Table S1. REMARK guideline features. [file HIS-86-226-s001.docx]

# SUPPLEMENTARY FILE


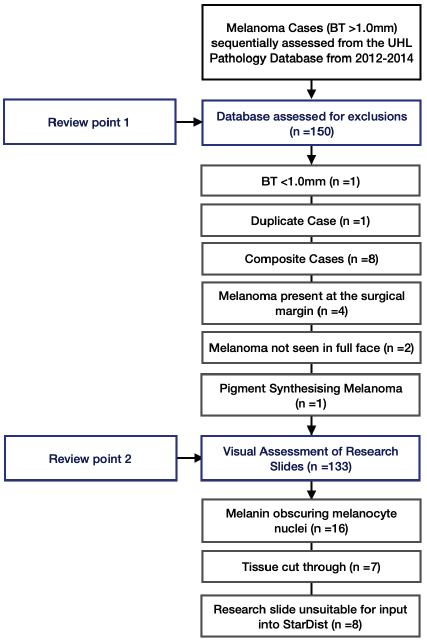


**Figure S1.** Selection of melanoma cases. Review point 1 entailed assessment of the original melanoma report and archival slides. Review point 2 entailed assessment of new sections that were cut and stained for the research study. Composite cases were those where the transverse was traversed more than one slide. This was to avoid image ‘stitching’ and ensuing artefacts. One slide was wrongly included in the database (BT < 1.0 mm). Melanoma extending to surgical margins or not seen in full face were excluded to avoid underestimate of the nuclear count. One pigment synthesising melanoma was excluded because nuclei were obscured. 16 were excluded because of intense melanin obscuring nuclei, 7 because tissue was cut through and 8 cases had weak SOX10 immunostaining or a tissue fold.


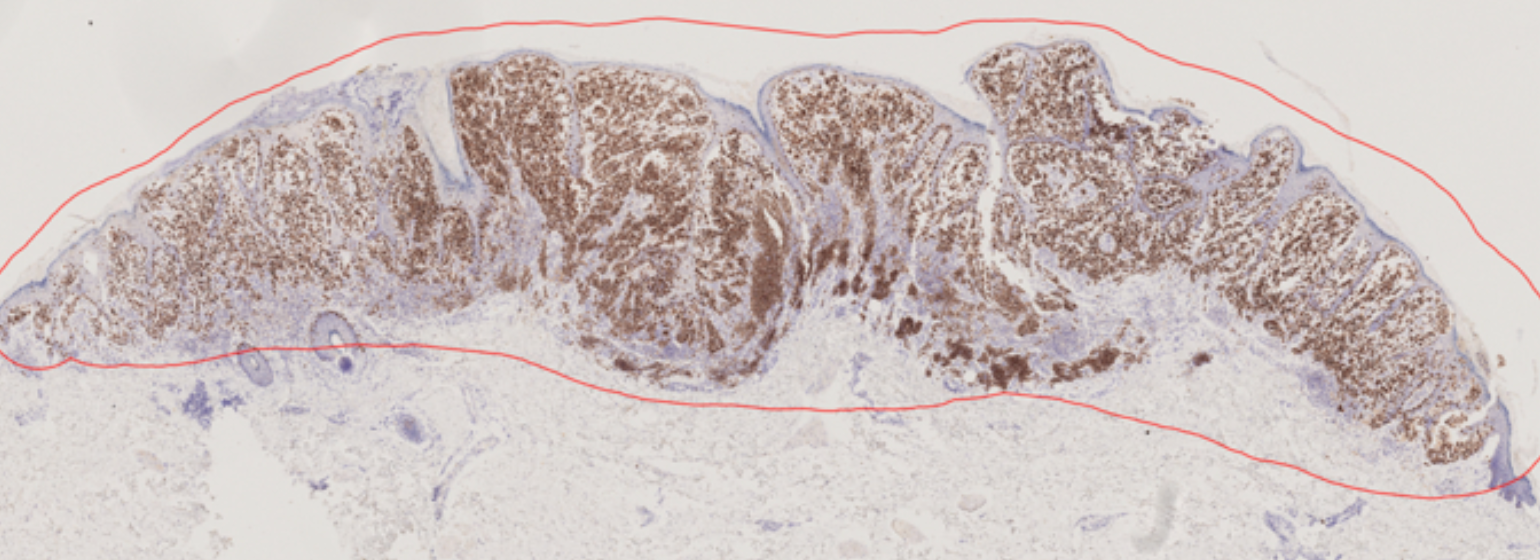


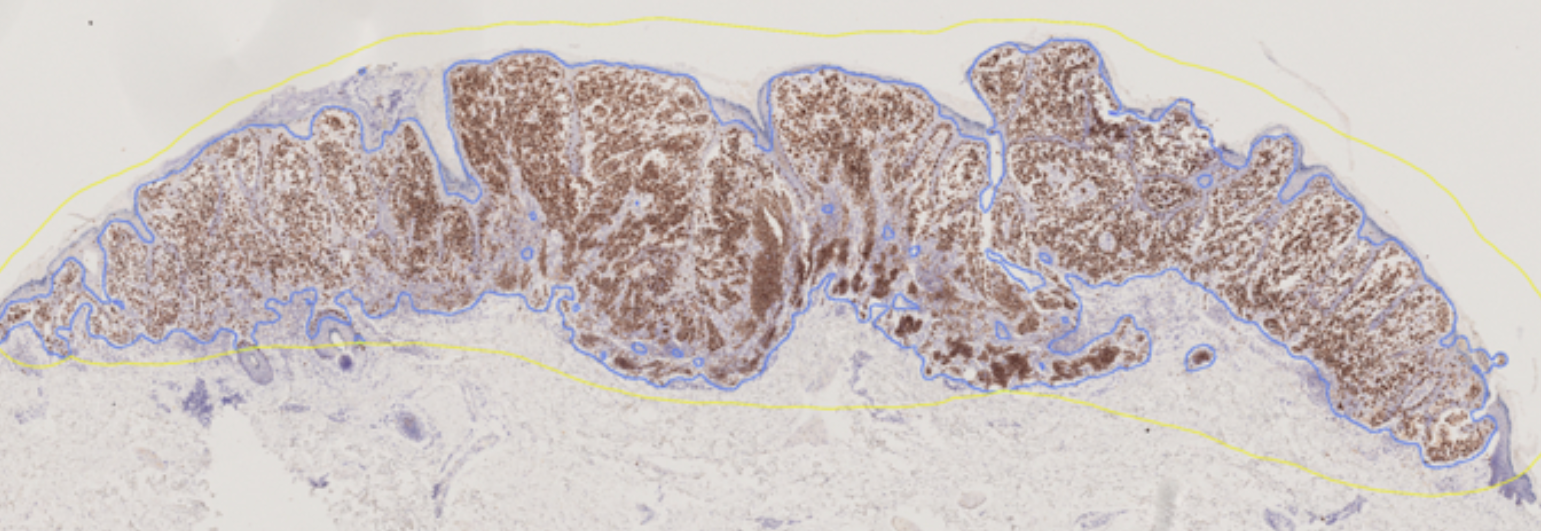


**Figure S2**. Top panel shows WSI with hand drawn selection in red. Lower panel shows the selections created by the pixel classifier in blue. This was then manually adjusted as necessary to remove in situ melanoma and non-tumour areas with melanin, typically in melanophages.

| **REMARK feature** | **Location in text** |
| --- | --- |
| 1. Biomarkers, objectives, and hypotheses | Introduction |
| 2. Patient characteristics | Methods |
| 3. Patient eligibility and inclusion/exclusion | Figure S1 |
| 4. Material used | Methods |
| 5. Assay methods | Methods, results |
| 6. Case selection | Figure S1, methods |
| 7. Study endpoints | Methods |
| 8. Candidate variables | Methods |
| 9. Sample size rationale | Methods |
| 10. Statistical methods | Methods and results |
| 11. Handling of marker values / cut points | Methods, results |
| 12. Flow of patients | Table S1 |
| 13. Demographic features | Table 1 |
| 14. Relation of marker to standard variables | Tables 2 and 3 |
| 15. Univariate analyses | Figure 4 and Table 2 |
| 16. Multivariable analyses | Table 2 |
| 17. Multivariable analyses including all variables | Table 2 |
| 18. Checking model assumptions | Methods |
| 19. Interpret results context; limitations of study | Discussion |
| 20. Implications for future research; clinical value | Discussion |

**Table S1 - REMARK guideline features**
